# Areas of modernization of the system of training and certification of forensic experts in Ukraine based on the adaptation of foreign experience

**DOI:** 10.1093/fsr/owae033

**Published:** 2024-07-02

**Authors:** Nataliia Martynenko

**Affiliations:** Department of Political Science and Law, Kyiv National University of Construction and Architecture, Kyiv, Ukraine; National Scientific Center “Hon. Prof. M.S. Bokarius Forensic Science Institute” of the Ministry of Justice of Ukraine, Kharkiv, Ukraine

**Keywords:** forensic sciences, qualification, automated anonymous testing, qualification examination, professional training

## Abstract

The article is devoted to the study of the existing system of training and certification of forensic experts in Ukraine and to the provision of proposals for its modernization, taking into account the positive experience in the field of forensic science of the USA, UK and some countries of the European Union. In Ukraine, the procedure for the training and certification of forensic experts is determined by ministries and other central executive authorities, which manage state specialized institutions that carry out forensic activities. A study of foreign experience in the training and certification of forensic experts made it possible to identify general and specific elements, sustain the benefits of the implemented measures, and formulate priorities and guidelines. The research resulted in the justification of the need to develop a single regulatory act that would determine the procedure for training, internships, and certification of forensic experts, as well as the creation of a single qualification body that would help ensure uniform approaches to determining the qualification of forensic experts working in state specialized forensic institutions, their territorial branches, expert institutions of municipal ownership, and forensic experts who are not employees of these institutions. It is proved that the development and implementation of uniform standards for training forensic experts on the basis of a centralized approach, regardless of where these specialists will work in the future, will help address the problem of training personnel. The author concludes that improving the quality of training, internship, and certification of forensic experts requires new approaches to organizational, economic, teaching, methodological, and technical provision. The author substantiates the need for fundamentally new forms of activity which will ensure the effectiveness and transparency of the assessment of knowledge by means of modern information technologies in the current reform environment. It is stated that Ukraine should raise the criteria for admission to the profession of a forensic expert. The author specifies the issues that need to be enshrined in law.

**Key points:**

- Implementation of uniform standards for the training of forensic experts will solve the problem of training unqualified personnel.
- Shortcomings in the system of training and certification of forensic experts lead to staff turnover in forensic institutions.
- Focusing the training on the individual needs of a forensic expert will improve the existing model of professional development.
- Establishment of a single institution for the qualification assessment of forensic experts will contribute to the formation of a unified practice of access to the profession.
- Establishment of the Institute of Forensic Expertise will facilitate the theoretical mastery of academic disciplines in the field of expert activity and the passage of relevant practices.

## Introduction

The professional training of a forensic expert is the basis for forensic science functioning. A forensic expert in any type of legal proceedings is a person knowledgeable in science, technology, art, craft, etc., and about objects, phenomena, and processes. The qualification of a forensic expert determines the scope and content of knowledge required to conduct expert examinations (investigations) of a particular kind or type, and the range of powers given by law. Competence is formed in the process of professional training. The qualification of a person is a determining factor in the admissibility of his or her involvement as a forensic expert and one of the main aspects of assessing the reliability of forensic examination results as evidence in court proceedings.

The competence of a forensic expert is formed by acquiring special knowledge determined by an expert specialty that is necessary for conducting expert examinations (investigations) of certain types (subtypes). The basis for the formation of expert specialties is the scientific classification of forensic examinations on the basis of their subject matter, object, and methodology. The combination of subject matter, objects, and methods of expert research constitutes a separate branch of special knowledge, which forms an independent class, kind, type, and subtypes of expertise and relevant expert specialties [1]. Specialized knowledge of a forensic expert should not be equated with knowledge of only a branch of science and technology that underlies the formation of a class, kind, type and subtype of forensic examinations, since without the knowledge of general scientific principles of forensic examination and its procedural support, it is impossible to properly conduct research and document its results so that the expert’s opinion is an admissible and reliable evidence. The professional training of a forensic expert allows the use of the acquired knowledge in legal proceedings and confirms the status of a forensic expert as a person who contributes to the administration of justice.

Modernization of the system of training and certification of forensic experts is currently one of the most pressing issues for forensic science in Ukraine. The purpose of modernizing this area is to create a mechanism for the sustainable development of the system of training and certification of forensic experts, to improve its quality, taking into account the balance of social interests and the market component.

Meeting the aim of upgrading the system of training, internships, and certification of forensic experts, and at the same time the quality of expert support for justice, requires a comprehensive approach. In this regard, it is advisable to turn to the experience of foreign countries in the development of this area of forensic science, in particular the USA, UK, Austria, France, Poland, and Romania.

### Literature review

A number of countries emphasize the changing needs of forensic science. Lack of funds and personnel lead to a decrease in efficiency and effectiveness, given the large number of applications for forensic examinations and expert investigation [2]. Funding for the education, training, and competency testing of forensic scientists will support laboratories in fulfilling their mission to assist the criminal justice system [3, 4]. In order to reduce staff turnover, attention should be paid to training current and future forensic laboratory personnel, using extended interviews and internships, and establishing links with universities to create new training programmes [5].

A survey of existing bachelor’s and master’s degree programmes in criminal science in the USA found that the surveyed forensic science programmes vary considerably in standards, primarily that concerns their size and subject matter, although most programmes provide a strong science curriculum, faculty with advanced degrees, and interesting forensic science-oriented courses [6]. A balance of theoretical and practical classes in the undergraduate curriculum is proposed, which will help reduce staff turnover and help solve the problem of staff shortages in forensic laboratories [7–11]. The introduction of an additional module to the core curriculum of the Bachelor of Medicine will help students to master knowledge and identify career opportunities in forensic science [12]. The content of the curricula should meet the needs of employers in the rapidly developing field of forensic science [13, 14]. During the survey, forensic laboratory managers and practitioners emphasized the benefits of specialized courses in specific disciplines in training the next generation of forensic scientists, while maintaining a strong foundation in the natural sciences at the undergraduate level [15].

Online continuing education is on the rise as this method of content distribution provides accessibility and convenience. Therefore, a model for developing, online delivery, and archiving of engaging and cost-effective continuing-education content for the forensic community has been proposed [16]. Physicians can acquire forensic skills at any point in their career as part of their continuing education in forensic medicine [17]. A survey of forensic practitioners concludes that mandatory certification of forensic experts will help establish competence in the profession and increase public confidence in forensic science [18].

The requirements of forensic laboratory employers have changed from a general, broad forensic science curriculum as described in current accreditation rules to a focused, subject-rich curriculum with additional management and professional content [15]. Managers of forensic laboratories are increasingly interested in initiating blind testing in at least some disciplines to verify the qualifications of forensic experts [19].

Ukrainian scholars state that the system of training personnel for the expert service of the Ministry of Internal Affairs of Ukraine does not meet the tasks currently facing the service [20]. The researchers believe that the system of training of expert personnel in the institutions of the Ministry of Justice of Ukraine should be aimed primarily at creating the most favorable conditions for the training of forensic experts, continuous improvement of their professional preparation by deepening and expanding professional knowledge, skills and abilities, as well as encouraging the transfer of accumulated knowledge [21].

The European Network of Forensic Science Institutes (ENFSI) Strategic Plan 2023–2026 emphasizes the need to identify and assess education, training, and proficiency testing needs [22]. Dissemination of information/training activities, such as e-learning of working experts and conducting qualification tests/collaborative exercises are important tools to improve the reliability, quality, and credibility of forensic science [23].

The use of information technology and the creation of appropriate software tools for conducting knowledge testing significantly increases the efficiency of the training process and is a promising area of the modern educational process. In our opinion, the problems of testing persons who have intention to obtain (confirm) the qualification of a forensic expert have received insufficient attention so far, which determines the relevance and importance of the present study.

In view of the above, the purpose of this article is to study the existing system of training and certification of forensic experts and to provide proposals for its modernization in Ukraine. For this purpose, it is planned to consider the positive experience of training and certification of forensic experts in the USA, the UK, and some countries of the European Union.

## Methods

To achieve the purpose of the scientific research presented in this article, the author used a systematic approach and a number of general scientific and special methods, in particular: comparative legal, functional, dialectical, comparative analysis, formal legal, synthesis, generalization, and prognostic.

The study used a systematic approach as one of the main areas of the methodology of special scientific knowledge and social practice, the purpose and objectives of which are to study certain objects as complex systems, taking into account the immediate needs of the subjects of forensic expert activity. The methodological specificity of the systematic approach is the study of patterns and mechanisms of a complex object formation based on numerous components, which allowed the author to develop recommendations for the introduction of a more effective model of training and certification of forensic experts in Ukraine.

With a view to reviewing the provisions of the current legislation of the countries under study regarding the training and certification of forensic experts, the author used the comparative legal method. The method allows studying the properties of legal phenomena, their similarities, and differences by comparing them in order to establish trends in their development. This made it possible to identify common and distinctive features in the training and certification of forensic experts and to suggest that positive foreign experience be implemented in Ukraine.

The functional method, as a general scientific method of cognition of complex legal phenomena and their regularities in a dynamic aspect, which determines the direction and shapes the way the cognitive process itself functions, and reflects the universal relations of the legal system, helped to outline the main areas of modernization of the system of training and certification of forensic experts in Ukraine.

The dialectical method of cognition, based on the cognitive potential of the categories of dialectics, which are the axiomatic foundations of any scientific worldview and universal logical forms of thinking, reflecting the properties, relations, and connections that exist in objective reality, accompanied the research process and provided an opportunity, in the context of the development of Ukraine’s legislation on forensic activity, to determine the directions of modernization of the system of training and certification of forensic experts and to provide proposals for improving the legal regulation.

Using the comparative analysis method, it became clear that in the USA, the UK, and France, the certification of forensic experts is carried out through testing, which makes it possible to quickly monitor and evaluate the quality of forensic expert training. Moreover, the method of comparative analysis helped to establish that monitoring the training of forensic experts provides an opportunity to understand and find ways and opportunities to improve its quality.

In analyzing the legislation of Ukraine and foreign countries in the area of research, the author uses the formal legal method, which is manifested in the analysis of sources of law, formal definiteness of law, the procedure for systematizing regulatory material, and the rules of legal technique. This method helped to clarify the content of the legal norms governing relations in the relevant area, as well as to develop recommendations for improving the current legislation of Ukraine on forensic experts training and certification.

The synthesis method helped to obtain new knowledge that the main purpose of the system of training and certification of forensic experts is to train qualified personnel for high-quality expert support of the justice system. In addition, this method made it clear that each of the countries studied is currently engaged in developing standards for the training of competent forensic experts.

The method of generalization helped to conclude that it is necessary to raise the criteria for admission to the forensic expert profession in Ukraine and to consolidate at the legislative level a number of provisions regarding the training of qualified personnel in order to ensure high-quality expert support of the justice system.

The prognostic method made it possible to predict the future benefits from creating a single qualification body that would help ensure uniform approaches to assessment of forensic experts’ qualifications, improve the quality of their training, internships, and certification.

## Results and discussion

According to Art. 16 of the Law of Ukraine “On Forensic Expertise” [24], “the purpose of certification of a forensic expert is to assess the professional level of specialists involved in conducting forensic examinations or participating in the development of the theoretical and methodological basis of forensic examination”, and “the purpose of certification of employees of a state specialized institution involved in conducting forensic examinations and/or participating in the development of the theoretical and scientific-methodological basis of forensic examination is to assess the level of their special knowledge and compliance with the position occupied” [24]. The qualification of a forensic expert giving the right to conduct a certain type of examination is assigned depending on the specialization.

In Ukraine, the procedure for certification and assignment or deprivation of qualification classes of a forensic expert is determined by ministries and other central executive authorities, which manage state specialized institutions that carry out forensic activities. The training of forensic experts is regulated by the legal acts of the Ministry of Justice of Ukraine, the Ministry of Health of Ukraine, the Ministry of Internal Affairs of Ukraine, the State Security Service of Ukraine, and the State Border Guard Service of Ukraine. In Poland, the Minister of Justice determines the procedure for the appointment of forensic experts, the performance of their duties and their dismissal [25].

**Figure 1:**
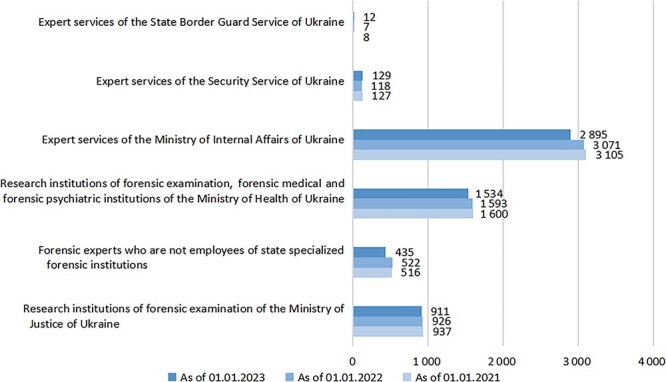
The number of certified forensic experts according to the State Register of Certified Forensic Experts in Ukraine. Source: compiled by the author based on [26].

The website of the Ministry of Justice of Ukraine contains information on the number of forensic experts who are included in the State Register of Certified Forensic Experts. As of 1 January 2023, the total number of forensic experts in Ukraine amounted to 5.92 × 10^3^ people [26] (Figure 1), with a population of 38 000.0 × 10^3^ people [27], i.e. 0.016%.

For comparison, in the USA, there were 18.5 thousand forensic experts for the same period [28], with a population of 333 287.56 × 10^3^ people, i.e. 0.006% [27]. In Poland, the total population is 37 561.6 × 10^3^ people [27], and the Search Engine of Forensic Specialists contains information on 15.66 × 10^3^ forensic experts [29], i.e. 0.04%. The List of Sworn and Court-Certified Experts and Interpreters in Austria includes 8.24 × 10^3^ forensic experts [30], with a total population of 9 042.53 × 10^3^ people [27], i.e. 0.09%. In France, the National Council of Companies of Justice Experts unites about 10.0 × 10^3^ forensic experts in >90 multidisciplinary regional expert companies [31] with a total population of 67 935.66 × 10^3^ people [27], i.e. 0.015%.

According to Art. 7 of the Law of Ukraine “On Forensic Expertise”, “forensic expert activity is carried out by state specialized institutions, their territorial branches, expert institutions of municipal ownership, as well as forensic experts who are not employees of these institutions, and other specialists (experts) in the relevant fields of knowledge in the manner and under the conditions determined by this Law” [24]. In the system of training, internships, and certification of forensic experts, on one hand, the consumer (a specialist who intends to obtain or confirm the qualification of a forensic expert) is looking for the opportunity of self-realization in the professional field of activity, and on the other hand, state specialized forensic institutions (employers) are looking for specialists who are able to perform professionally their service duties and quickly adapt to the working conditions. According to Art. 10 of the Law of Ukraine “On Forensic Examination”, “forensic experts may be persons who have the necessary knowledge to provide an opinion on the issues under investigation” [24], and “forensic experts of state specialized institutions may be specialists who have the appropriate higher education, educational qualification level not lower than a specialist, have received an appropriate training, and were awarded the qualification of a forensic expert in a particular specialty” [24].

The Order of the Ministry of Justice of Ukraine defines the procedure for training and internship of specialists who intend to obtain or confirm the qualification of a forensic expert, and the procedure for certification for the purpose of assigning or confirming the qualification of a forensic expert at the Central Expert Qualification Commission under the Ministry of Justice of Ukraine [32]. In order to assign or confirm the qualification of a forensic expert, employees of forensic research institutions and specialists who are not employees of state specialized forensic institutions must have a master’s degree, receive training (internship), and know the theoretical, organizational, and procedural issues of forensic examination and the methodological provisions and practice of their application in the relevant expert specialty.

It is worth noting that the Decree of the Minister of Justice of Poland “Regarding Forensic Experts” paragraph 12 contains the requirement that a forensic expert must be “over 25 years old”, “possess theoretical and practical special knowledge in the field of science, technology, art, craft, as well as other skills”, which is to be confirmed by documents or other evidence evaluated by the president of the relevant court [33]. French legislation requires that an individual may be registered or re-registered in the list of experts provided that he or she is under 70 years of age [34]. According to the Romanian legislation, the requirements for registration for participation in the competition for a vacant position of a forensic expert include the following: a bachelor’s degree; knowledge of a foreign language of international communicationat an intermediate level [35, 36]. In the USA, forensic specialists are usually required to have at least a bachelor’s degree with further on-the-job training [28].

The Qualification Chamber of the Central Expert Qualification Commission under the Ministry of Justice of Ukraine decides on the professional training and conducts certification to determine the level of training of the staff of forensic research institutions and specialists who are not employees of state specialized institutions, and considers the issue of assigning a qualification class of forensic expert.

Training and internships are carried out by forensic research institutions that have the appropriate license to carry out educational activities in the field of advanced training. They may engage forensic experts who are not employed by state specialized forensic institutions but have at least 3 years of practical experience as a forensic expert.

The training and internship programmes published on the official website of the Ministry of Justice of Ukraine provide for lectures and practical classes, preparation of draft opinions and their review [37].

The minimum volume of the training programme in case of awarding the qualification of a forensic expert is 40 academic hours (hereinafter referred to as Programme No. 1). In the case of the first confirmation of the qualification of a forensic expert (hereinafter referred to as Programme No. 2), and subsequent confirmations of the qualification of a forensic expert (hereinafter referred to as Programme No. 3), the volume of the programme is reduced by 1/3 of the volume of the previous programme.

A specialist who intends to obtain the qualification of a forensic expert for the first time is trained under a training programme on theoretical, organizational, and procedural issues of forensic examination, as well as under training programs in the relevant expert specialties. Reviewing of expert conclusions in each expert specialty is provided by research institutions of forensic examinations and forensic experts who are not employees of state specialized forensic institutions.

Specialists who are not employees of state specialized forensic institutions and intend to obtain or confirm the qualification of a forensic expert pay the relevant institutions training and internship fees, the amount of which is determined in accordance with the cost estimate, taking into account the number of training hours for lectures, practical classes, and the number of hours for reviewing draft opinions [38].

Using the example of a forensic economic examination, consider the cost of training (internship) in two research institutes of forensic examination (Table 1) [39, 40]:

**Table 1:** The cost of training (internship) in two forensic research institutions. Compiled by the author based on [39, 40].

The cost of training (internship) for three types of expert specialty 11.1, 11.2, 11.3	Kyiv Scientific Research Institute of Forensic Expertise (KSRIFE)	Оdesa Scientific Research Institute of Forensic Expertise (OSRIFE)
Programme No. 1	UAH 65 300.40 (21 766.80 × 3)	UAH 77 173. 20 (25 724.40 × 3) UAH 92 336.40 (30 778.80 × 3) if the internship supervisor is a PhD in Economy
Programme No. 2	UAH 43 335.72 (14 445.24 × 3)	UAH 61 738.56 (20 579.52 × 3) UAH 73 869.12 (24 623.04 × 3) if the internship supervisor is a PhD in Economy
Programme No. 3	UAH 28 494.72 (9 498.24 × 3)	UAH 46 303.92 (15 434.64 × 3) UAH 55 401.84 (18 467.28 × 3) if the internship supervisor is a PhD in Economy

At Odesa Scientific Research Institute of Forensic Expertise, the cost is higher due to the greater number of training hours for lectures and practical classes. The cost of training is also affected by the fact that the supervisor of the internship may be a forensic expert with a scientific degree.

Certification is carried out by conducting a qualification examination in oral form—answering the questions posed in the examination papers. Examination papers for testing the knowledge of theoretical, organizational, and procedural issues of forensic examination are based on the training programme and contain two questions; those for testing knowledge of methodological provisions in the relevant expert specialties—three questions. The result of the qualification examination is evaluated as “passed” or “failed”.

In case of a decision to award or confirm the qualification of a forensic expert, on the day of the qualification examination a certificate of qualification as a forensic expert in a certain type of expert specialty is issued, or a note is made on the extension of its validity, and the information is entered into the State Register of Certified Forensic Experts. In France, according to Art. 6 of the Law on Forensic Experts, when registering for the first time on the list, experts take an oath before the Court of Appeal, undertaking to fulfill the mission, to report and express their opinions in their reports, to be honest and fair [41]. Similarly, paragraph 4 of the Regulation of the Minister of Justice of Poland “On Forensic Experts” stipulates that before assuming his/her duties, an expert shall take an oath to the president of the relevant court as follows: “Realizing the importance of my words and my responsibility before the law, I solemnly promise that I will perform my duties as a forensic expert with full integrity and impartiality” [33].

In Poland, according to Article 157 of the Law on the System of General Courts, “the president of the regional court appoints court experts and keeps a list of them” [25]. An expert included in the list of court experts must have an account on the information portal.

Persuant to the provisions of the French Decree on Forensic Experts, the initial registration of a forensic expert in the lists of the Court of Appeal is carried out on the basis of an application submitted by 1 March of the current year for the following year at the place of professional activity or residence [34]. Initial registration in the list of the Court of Appeal is made on a trial basis for a period of 3 years, after which the experience of the person concerned and the acquisition of legal knowledge are assessed. Subsequently, each re-registration is carried out for a period of 5 years based on the opinion of a commission consisting of judges and experts. Registration on multiple lists is prohibited. Experts registered in the list of the Court of Appeal may apply for registration in the National List by 1 March by contacting the Court of Cassation.

In Romania, a forensic expert of the National Institute of Forensic Expertise is a civil servant who is hired for a vacant position on a competitive basis [42]. The competition is announced by specialties of expertise, taking into account the needs of the National Institute of Forensic Expertise [35].

In the UK, forensic training is provided in undergraduate and graduate courses at universities and colleges and covers a wide range of disciplines. In addition to academic courses, there are also vocational training programmes that provide practical training in forensic methods [43].

In most countries, when recruiting employees to forensic institutions, preference is given to university graduates with a master’s degree in forensic science. In accordance with the independent EduRank ranking, the top 100 forensic universities include 29 universities in the UK, 19 in Germany, 10 in Italy, 7 in the Netherlands, 6 in Sweden, 6 in Spain, 3 in Austria, 3 in Poland, etc. [44]. The British University of Leicester ranks there first.

The Order of the Ministry of Health of Ukraine regulates the procedure for certification for assigning or confirming the qualification of a forensic expert by expert qualification commissions operating at institutions providing forensic examinations [45]. The certification is carried out by conducting a qualification examination—oral answers to questions posed in the examination papers. During the examination, a person should demonstrate a satisfactory knowledge of theoretical, organizational, and procedural issues of forensic examination and methodological provisions of the relevant forensic medical expert specialties. The result of the qualification examination is assessed as “passed” or “failed”. In case of a decision is made to award or confirm the qualification of a forensic expert, a person is issued a certificate of qualification as a forensic expert in a certain type of expert specialty within 30 working days. The head of the institution must submit a request to the structural unit of the Ministry of Justice of Ukraine responsible for the organizational and managerial support of forensic activities to enter information into the State Register of Certified Forensic Experts regarding: the issuance of a certificate of qualification of a forensic expert, or the inclusion of a note in the certificate on the extension of its validity.

In Romania, the criteria for the certification of a forensic expert are developed by the Supreme Council of Forensic Medicine and approved by an order of the Minister of Health [46]. An examination commission of three members of the Higher Council of Forensic Medicine conducts an examination on knowledge of the current procedural, medical legislation, and rules of professional conduct [47]. The list of forensic experts is published on the official website of the Romanian Society of Forensic Medicine and is brought to the attention of the judiciary.

In France, after obtaining a master’s degree in medical sciences, a candidate passes national ranking tests that allow them to choose a specialty and undergo an internship [48].

The Order of the Ministry of Internal Affairs of Ukraine defines: the procedure and conditions for awarding the qualification of a forensic expert in a certain type of forensic examination and expert specialty to the staff of the Expert Service of the Ministry of Internal Affairs of Ukraine who hold positions that involve organization and conduct of forensic examinations and are trained for the purpose of certification as forensic experts; the procedure and conditions for awarding the qualification class of a forensic expert; the procedure for organizing and conducting training and advanced training; the procedure and conditions for certification of forensic experts to assess their professional level and determine their suitability for the position held [49].

The personnel of the Expert Service of the Ministry of Internal Affairs of Ukraine is trained according to the forensic expert training programmes in the relevant expert specialties approved by the Chairman of the Expert Qualification Commission of the Ministry of Internal Affairs of Ukraine, through individual training, internships, or specialty-tailored courses. It is allowed to complete several types of training simultaneously. The period of basic training should not exceed 3 months, in some fields—6 months. The period of additional training shall not exceed 1 month, and for certain programmes—2 to 3 months. After completing the training, assigning the qualification of a forensic expert to employees is decided upon the results of the qualification examination taken orally. Examination papers for testing the level of knowledge of the provisions of forensic examination laws, procedural law, and special training include five questions on the training programmes for forensic experts of the relevant expert specialty and one practical task. The qualification of a forensic expert is awarded if the final grade is “excellent”, “good”, or “satisfactory”, which is the basis for inclusion in the State Register of Certified Forensic Experts.

The National Scientific Police Service, established under the French National Police Directorate General, recruits and selects, trains, and develops personnel [50]. There are two competitions: an internal competition for civil servants, and an external competition for persons who are not in the civil service. The tests are identical for both competitions (external and internal). The competition is held in two stages: qualification tests and entrance tests. The qualification tests include three stages that last 2 h each: multiple choice questions—the candidates answer several questions and solve problems; comprehension tests—the candidates answer 6 to 8 questions based on the results of reading a text of ~350 words—used to assess the candidates’ ability to understand, interpret, and organize basic ideas; psychotechnical tests that assess the candidates’ psychological profiles, taking into account professional requirements. The entrance test consists of an oral interview, during which the candidates make 5-min presentations, and then answer the questions of the commission for 15 min, and a foreign language test—a 15-min discussion in English, Spanish, German, or Italian—according to candidates’ preferences [51]. The commission summarizes the scores obtained in the four tests (multiplied by their coefficients) to obtain the total number of points received by the candidates in the competition. After being appointed as trainees, the candidates take several theoretical courses and a practical internship during the year [52].

In the UK, it is possible to get a job as a forensic expert in the police by passing an online test and an interview with a presentation in the chosen field of work [53].

The Order of the Security Service of Ukraine defines the procedure for certification of forensic experts of the Security Service of Ukraine for awarding or confirming the qualification of a forensic expert and/or assigning a qualification class of a forensic expert [54]. Training and internships of forensic experts are carried out in accordance with the training plans for the relevant expert specialties, which are approved by the head of the expert qualification commission of the Security Service of Ukraine. After completion of the training course, in order to assess the quality of specialists’ training and the amount of methodological material they have learned, they are interviewed, and their answers to the questions posed by the members of the commission are evaluated. A specialist is notified of the results of the certification within 10 days and, in case of awarding the qualification of a forensic expert or confirming the previously awarded qualification of a forensic expert, the information is entered into the State Register of Certified Forensic Experts.

At the Central Intelligence Agency’s (CIA) Directorate of Science and Technology, most entry-level positions require a bachelor’s degree, but expert-level positions typically require of candidates to have both a degree and significant work experience [55]. The requirements for the position of a forensic expert include: an advanced degree, formal training in a forensic discipline, experience as a forensic professional and technical analyst, experience of work in an accredited and recognized forensic laboratory, familiarity with forensic standards and requirements, current membership in recognized professional organizations within the field, strong verbal presentation skills, ability to write clear, concise text, etc. [56]. Based on the results of the review of the submitted resume, it is proposed to apply for a certain position, after which it is necessary to successfully pass the selection, testing, and interview.

Attention should be paid to the practice of the CIA’s Directorate of Science and Technology of selecting candidates through student programmes—they offer “financial needs-based scholarships for undergraduate and graduate students. In addition to a year-round salary, scholarship recipients get up to USD25 000 in tuition assistance per calendar year” [57]. However, there are certain conditions: “undergraduate students must work at least one, and preferably two, 90-day session(s) at CIA before graduating; graduate students must work at least one 90-day tour at CIA before graduating; after graduation, all scholarship recipients must work at CIA for a period of 1.5 years per year of paid scholarship received” [57].

The Regulation on the Expert Qualification Commission and Certification of Forensic Experts of the State Border Guard Service of Ukraine was approved by the order of the Administration of the State Border Guard Service [58]. Training and internships of specialists are carried out according to the programmes for training forensic experts in the relevant expert specialties, which are approved by the Chairman of the Expert Qualification Commission of the State Border Guard Service of Ukraine and include lectures, seminars, practical classes, and drafting of opinions provided for review. The training period for awarding the qualification of a forensic expert is set depending on the type of expert specialty, the level of basic training, and the specialist’s experience in the relevant field of knowledge; as a rule it is 3 months. The certification includes consideration of the submitted documents and a qualification examination, during which experts answer the questions posed in the examination paper on procedural law, special training, and the use of technical and forensic means in conducting expert examinations and investigations. The specialist is notified of the results of the certification within 10 days. Specialists who have been awarded or confirmed the qualification of a forensic expert are included in the State Register of Certified Forensic Experts.

In the UK, after being recruited by the Border Agency, which is part of the Home Office, applicants take a structured training program, usually consisting of three parts: pre-course learning, classroom learning, mentoring. Training in immigration and customs law is modular and requires testing throughout the program duration [59]. After completing the training, a probationary period is established. Beginners are provided with mentoring assistance from an experienced Border force officer to help them practice the material they have learned [60].

When applying to the US Customs and Border Protection, candidates are evaluated based on their resume, supporting documents, and a two-part entrance exam: (i) an online assessment and (ii) a supervised exam administered at a local testing center that lasts 4 h and includes the following three assessments: Logical Reasoning, Arithmetic Reasoning, and Writing Skills. Applicants must pass the exam with a score of at least 70 [61]. Employment verification can last up to 12 months, or longer [62].

The results of the analysis of regulatory legal acts of the Ministry of Justice of Ukraine, the Ministry of Health of Ukraine, the Ministry of Internal Affairs of Ukraine, the Security Service of Ukraine, and the State Border Guard Service of Ukraine show that the training of forensic experts is carried out in the form of additional professional education in a specific expert specialty for specialists who have the appropriate higher education with educational qualification level not lower than a master’s degree. This form of forensic expert instruction is implemented as an additional training with the mastery of procedural law and the theoretical and methodological foundations of forensic examination. It is based on a mentoring system and is considered effective, since under the guidance of experienced and qualified mentors it allows trainees to master the scientific, methodological and instrumental foundations, learn to draft expert opinions, and receive the necessary assistance and advice. In the course of training, a future forensic expert is gradually introduced to the profession by acquiring the necessary knowledge and practical skills. Upon completion of additional professional training, he or she may be admitted to certification for the right to conduct an independent forensic examination. However, it should be noted that premature admission to the profession is fraught with expert errors and may adversely affect justice outcomes. Therefore, the training should be carried out according to an individual plan, which is drawn up depending on the level of education, characteristics, and needs of each person.

It is important to understand that an expert’s qualification is limited to the list of issues in which he or she is knowledgeable. While objective competence refers to the amount of knowledge that an expert should possess, subjective competence refers to the knowledge that he or she actually possesses. This means that the competence of an expert can be either wider than the required objective competence for the production of an expertise or narrower, in which case the expert will be considered incompetent, i.e. not having enough specialized knowledge for the production of an expert opinion. The worst case is when an expert has only limited competence in the matter under consideration, which can lead to false confidence of those who assigned the examination in the expert’s competence in this area, although in fact the expert does not have sufficient knowledge.

The analysis of bylaws made it possible to see the problem situation of discrepancies between the systems of training, internships, and certification of forensic experts in different agencies of Ukraine, which have developed their own approaches to the activities of specialized forensic institutions under their jurisdiction. The current system of training and certification of forensic experts has certain shortcomings, which results in staff turnover in forensic research institutions.

Forensic examination is an investigation conducted by a forensic expert on the basis of special knowledge in the field of science, technology, art, craft, etc. of tangible and intangible objects, phenomena and processes, which contain information about the facts and circumstances of the case that is or will be the subject of litigation [63]. Therefore, the training and internship of specialists who intend to obtain or confirm the qualification of a forensic expert, as well as certification for the purpose of assigning and confirming the qualification of a forensic expert, is an important aspect of the justice system.

Today, the community of forensic experts in Ukraine has no qualification rules or a single self-regulatory organization that would present a strategic vision for the development of forensic science. Establishing a single institution for the qualification assessment of forensic experts will contribute to the formation of a unified practice of access to the profession.

In this regard, the experience of the UK should be taken into account, where the quality of forensic experts’ training is controlled by the Forensic Science Regulator [64] and the Chartered Society of Forensic Sciences [65], which ensure that forensic experts meet high standards of professionalism, competence, and ethical behavior. It is worth mentioning that in France forensic experts from >90 multidisciplinary regional expert companies are united by the National Council of Companies of Justice Experts [66], incorporating a Commission for Training and Quality of Expertise which has developed, with account of changes in legislation and regulations, and maintains 35 copyrighted training modules, available only to company executives and commission members [67].

The Order of the Ministry of Justice of Ukraine envisages creation of an electronic system for automated anonymous testing of persons wishing to obtain or confirm the qualification of a forensic expert. It is intended to provide the computer equipment of the Ministry of Justice of Ukraine with the system for conducting qualification examination and a supply of test tasks [68]. This order, introducing an electronic system for automated anonymous testing of persons who have expressed an intention to obtain or confirm the qualification of a forensic expert, comes into force 1 year after the date of termination or lifting of martial law [69, 70].

The automated anonymous testing will consist of three stages, which involve: test tasks to check the knowledge of theoretical, organizational, and procedural issues of forensic examination; test tasks to check the knowledge of issues within the expert specialties; a situational assignment proposed by the testing system.

It should also be noted that the preparation of proposals for test tasks in order to formulate test questions on theoretical, organizational, and procedural issues of forensic examination, issues within expert specialties, as well as the formation of situational tasks for the qualification examination to certify forensic experts was entrusted to the Scientific Advisory and Methodological Council on Forensic Expertise under the Ministry of Justice of Ukraine [71].

Tests are technologically effective and are a convenient and efficient method of controlling and assessing the quality of forensic experts’ training. Unlike the qualification examination, which is conducted orally by answering the questions posed in the examination papers, testing is an objective, comprehensive, humane, broad, and highly effective way of evaluation. The objectivity of testing is achieved by standardizing the procedure, and the impossibility of introducing a subjective component into the assessment of the knowledge of specialists who intend to obtain or confirm the qualification of a forensic expert.

However, other ministries and state bodies, which manage state specialized forensic institutions, have not yet considered the issue of introducing testing of persons who have expressed an intention to obtain or confirm the qualification of a forensic expert.

## Conclusions

In Ukraine, there is a need to develop a single regulatory act that will determine the procedure for training, internships, and certification of forensic experts, as well as to create a single qualification body that would help ensure uniform approaches to determining the qualification of forensic experts working in state specialized forensic institutions, their territorial branches, expert institutions of municipal ownership, and forensic experts who are not employees of these institutions. The development and implementation of the unified standards for the training of forensic experts based on a centralized approach, regardless of where the expert will work in the future, will help to solve the problem of training personnel.

According to the results of the study, taking into account foreign experience, it is necessary to state that Ukraine should raise the criteria for admission to the profession of a forensic expert, namely:

- to fix at the legislative level the age limit for awarding forensic expert qualification or confirmation of the previously awarded qualification and being in the status of a forensic expert;
- to establish requirements for the number of years of professional experience after obtaining a higher education degree, preceding admission to the profession of a forensic expert;
- to enshrine in the legislation the requirement for forensic experts’ proficiency in the official national language in accordance with the standard determined by the National Commission for State Language Standards;
- to make the qualification exam more difficult and conduct it in several stages: competitive selection, testing, and interview;
- to introduce tests to determine psychological readiness, speed of thinking, and communication skills to assess the candidate’s psychological profile in relation to the requirements of the profession;
- to introduce tests to assess candidates’ intellectual development: verbal, logical, abstract thinking, and the ability to write a clear and concise text;
- to introduce an exam for proficiency in a foreign language that is one of the official languages of the Council of Europe.

Improving the quality of training, internships, and certification of forensic experts calls for new approaches to organizational, economic, teaching, methodological, and technical provision. Therefore, there is a need for fundamentally new forms of activity that will ensure the effectiveness and transparency of the assessment of knowledge of individuals by means of modern information technologies in the current reform environment.

A list of test questions on theoretical, organizational, and procedural issues of forensic examination, questions within expert specialties and situational tasks should be developed. It is necessary to create and configure an electronic system for automated anonymous testing of persons who have expressed an intention to obtain or confirm the qualification of a forensic expert.

Forensic experts contribute to the administration of justice, and therefore there is a need to enshrine in law such issues as:

- acquiring the status of a forensic expert solely upon certification, qualification, and taking an oath, with the subsequent entry of this information into the Register of Certified Forensic Experts of Ukraine;
- provision of mentoring assistance from more experienced forensic experts to master the studied material in practice—practical training of a person after passing the qualification exam before he or she can fully work as a forensic expert;
- sending employees to study for a third degree (Doctor of Philosophy, PhD) in the relevant higher education institutions in accordance with their expert specialty.

It is advisable to organize professional orientation of young specialists as to mastering expert specialties and to adapt future employees to expert activities.

The existing model of advanced training of forensic experts should be upgraded through increasing its frequency, improving its quality, focusing the training on experts’ individual needs, and providing a stricter monitoring.

We deem it expedient to create the Institute of Forensic Science in order to provide forensic science students already at the bachelor’s level with an opportunity to study the theory and communicate directly with professionals working in various forensic specialties. This will facilitate both the mastery of theoretical academic disciplines, including those related to the profile of expert activity, and the completion of the relevant internships.

## Compliance with ethical standards

This article does not contain any studies with human participants or animals performed by the authors.

## Disclosure statement

None declared.
